# Current Insights into the Role of Peripheral Blood Immune Cell Phenotypes in Resistance to Cancer Therapies

**DOI:** 10.32604/or.2026.079865

**Published:** 2026-09-14

**Authors:** Allinson Olaechea, Cristina Camacho Rubio, Sara Gómez-Melero

**Affiliations:** 1 Department of Oral Surgery and Implant Dentistry, University of Granada, Granada, Spain; 2 Instituto de Investigación Biosanitaria, IBS.GRANADA, Granada, Spain; 3 Maimonides Biomedical Research Institute of Cordoba (IMIBIC), Reina Sofia University Hospital, University of Cordoba, Cordoba, Spain; 4 Department of Biochemistry and Molecular Biology, University of Cordoba, Cordoba, Spain

**Keywords:** Cancer, immune phenotype, peripheral blood mononuclear cells (PBMCs), immunotherapy, prognosis, immune checkpoints

## Abstract

Peripheral blood mononuclear cell (PBMC) immunophenotyping has emerged as a promising non-invasive approach to characterize systemic immune alterations in cancer and to identify biomarkers associated with treatment response and resistance. However, current evidence remains fragmented and predominantly descriptive, with substantial heterogeneity in study design, immunophenotyping methodologies, and patient populations, limiting the identification of robust and clinically translatable immune signatures. In this review, we aim to comprehensively analyze PBMC immune phenotypes across multiple cancer types, with particular emphasis on their association with disease progression, therapeutic outcomes, and the key methodological and translational challenges that currently limit their clinical implementation. Across malignancies, conserved immune features are consistently observed, including T cell exhaustion, regulatory T cell (Treg) expansion, and upregulation of immune checkpoint molecules, reflecting chronic immune activation and dysfunction. In parallel, tumor-specific phenotype, such as peripheral helper T (Tph) cell expansion in non-small cell lung cancer, Vδ1^+^CD69^+^ γδ T cells in hepatocellular carcinoma, and T-cell immunoreceptor with Ig and ITIM domains (TIGIT) positive dysfunctional T cells in oral squamous cell carcinoma, highlight the influence of tumor-specific immune contexts. Importantly, this review moves beyond descriptive reporting by integrating mechanistic insights into how PBMC phenotypes contribute to therapeutic resistance. Key mechanisms include immunosuppressive cytokine signaling (e.g., interleukin-10 (IL-10), transforming growth factor-β (TGF-β)), chronic antigen stimulation driving T cell dysfunction, and systemic immune-tumor crosstalk mediated by chemokine axes such as stromal cell-derived factor 1 (SDF-1)/C-X-C motif chemokine receptor 4 (CXCR4). In addition to lymphoid populations, we emphasize the contribution of myeloid cell subsets, including monocytes and myeloid-derived suppressor cells, as central regulators of immune evasion and treatment failure. Despite these advances, significant challenges remain, including the lack of standardized protocols, limited longitudinal and multicenter validation studies, and insufficient integration of multi-omics approaches. Addressing these limitations will be essential for clinical translation. Overall, this review provides a refined conceptual framework that distinguishes conserved and tumor-specific immune signatures and highlights their mechanistic relevance in therapeutic resistance, supporting the development of PBMC immunophenotyping as a tool for personalized cancer immunotherapy.

## 1 Introduction

In the immune system, the fundamental function of immune cells is to patrol the entire organism to distinguish malignant or premalignant cells from normal ones and eliminate them before they can cause harm. This phenomenon, known as *cancer immunosurveillance*, constitutes the basis of cellular immunotherapy in cancer [1,2,3,4]. Such immunotherapy aims to reactivate or enhance the patient’s own immune system through a series of mechanisms designed to selectively target cancer cells [5,6]. Although conventional treatments such as surgery, chemotherapy and radiotherapy remain the first-line therapies for most malignancies, immune cell-based therapies have emerged as a powerful complementary alternative for cancer patients [1,7,8].

Despite the promising results of immunotherapeutic approaches, the rates of clinically significant adverse events remain high and are often associated with absent or suboptimal immune responses. Several studies have investigated the immunophenotype of peripheral blood mononuclear cells (PBMCs) to address deviations in patient profiles compared with those of healthy donors, aiming to identify critical cellular subsets associated with therapeutic response or resistance. The immunophenotypic profiles of PBMCs have been linked to distinct immune modulations with prognostic and predictive relevance [9,10].

Early cancer detection is crucial for improving patient outcomes, and the immune system plays a key role in tumor development and progression [11]. PBMCs are considered a major source of immune cells that infiltrate tumors [12], undergoing a gradual transformation toward more immunosuppressive and migratory phenotypes. In addition to providing novel prognostic and predictive biomarkers, the characterization of PBMCs may have important implications for the development of innovative therapeutic strategies based on combined immunotherapeutic approaches [13].

A better understanding of T-cell immunity within the PBMCs of cancer patients could lead to a more rational approach to immunotherapy [14]. It is important to determine whether the cellular phenotype reflects T-cell functionality and whether it is associated with molecular cancer subtypes or prior treatments. Regardless of whether these changes are driven by the disease or by treatment, the resulting immune profile represents a relevant context for the development and optimization of cancer immunotherapies [15].

In addition to T cell subsets, myeloid cell populations within PBMCs, including monocytes, dendritic cells, and myeloid-derived suppressor cells (MDSCs), play a critical role in shaping systemic immune responses in cancer. These cells contribute to tumor progression and therapeutic resistance through antigen presentation defects, secretion of immunosuppressive cytokines, and modulation of T cell activation [16].

In recent years, the role of immune checkpoint molecules has emerged as one of the most promising avenues for biomolecular and clinical discovery. Immune checkpoints can reduce T-cell function or even deactivate them through a series of cascading effects [5]. These molecules include both stimulatory and inhibitory components, and their expression can reveal patterns of T cell and natural killer (NK) cell dysfunction or exhaustion [17]. Some of the first immune checkpoints identified were cytotoxic T-lymphocyte–associated antigen 4 (CTLA-4), programmed cell death protein 1 (PD-1), and programmed death-ligand 1 (PD-L1) [5]. However, many patients still fail to respond to therapies targeting conventional immune checkpoints, making it crucial to identify additional functional immune checkpoints. Consequently, novel immune checkpoints such as T-cell immunoglobulin and mucin-domain containing-3 (TIM-3), lymphocyte-activation gene 3 (LAG-3), and T-cell immunoreceptor with Ig and ITIM domains (TIGIT) have emerged [18,19,20,21].

The ability to modulate these molecular regulators has introduced a new paradigm in targeted cancer therapy [17]. Immune checkpoint inhibitors (ICIs) can block the function of these checkpoints, reactivate the antitumor effect of tumor-infiltrating T cells (TILs), and reshape immune function to treat cancer and prolong patient survival [5,18,22,23]. Encouragingly, immune checkpoint blockade therapy has transformed the cancer treatment landscape, producing more durable responses than targeted therapy or chemotherapy in certain tumor types [24].

The expression of immune checkpoint receptors on PBMC-derived lymphocytes from cancer patients may reflect the profile of these cells within the tumor. Moreover, their analysis could serve as a non-invasive method to predict treatment response [17]. Identifying immune parameters associated with pathological response may allow for better patient selection through biomarkers or help uncover mechanisms of treatment resistance. Across multiple tumor types, PBMC analysis has revealed alterations in immune cell populations and checkpoint molecules expression in response to systemic therapy [25,26]. Thus, the study of immune cells provides valuable insights into the mechanisms of response and resistance in immunotherapy [13].

The immune response to cancer is highly complex and plays a central role in disease progression and prognosis. The influence of tumors on the immune system occurs not only within the tumor microenvironment (TME) but also extends to the peripheral blood. The immune system, and particularly cellular immunity, may play a crucial role in tumor protection [12].

Therefore, the purpose of this review is to provide an updated and integrative analysis of PBMC immunophenotypes across different cancer types, with particular emphasis on their association with disease progression, therapeutic resistance, and clinical outcomes. In addition, this review aims to discuss the potential translational relevance of PBMC-based immune biomarkers and the current challenges limiting their clinical implementation.

## 2 Methods

This work was designed as a narrative review aimed at providing a comprehensive and up-to-date overview of peripheral blood immune cell phenotypes associated with resistance to cancer therapies.

A structured literature search was conducted across multiple databases, including PubMed, Scopus, and Web of Science, covering studies published up to 10/2025. The search strategy combined keywords related to peripheral blood immune profiling and cancer therapy resistance, including but not limited to: “PBMC”, “peripheral blood mononuclear cells”, “immunophenotype”, “immune profiling”, “cancer”, “tumor microenvironment”, “therapy resistance”, and “immunotherapy”.

Studies were selected based on their relevance to (i) immune phenotyping of peripheral blood cells, (ii) association with cancer progression or treatment response, and (iii) translational or clinical implications. Both solid and hematological malignancies were considered. Priority was given to recent studies, original research articles, and reports including human patient data.

Given the heterogeneity of study designs, cancer types, and immune markers evaluated, a formal meta-analysis was not performed. Instead, findings were qualitatively synthesized to identify consistent patterns, emerging biomarkers, and potential mechanistic insights across studies.

To minimize selection bias, we aimed to include representative studies across different cancer types and immune cell populations. When appropriate, conflicting findings and study limitations were explicitly discussed.

Preprints and gray literature were not included and emphasis was placed on peer-reviewed publications.

Overall, this approach allows for an integrative and critical appraisal of current evidence while acknowledging the limitations inherent to narrative reviews.

## 3 Cell Phenotypes in Cancer Patients

Several studies have reported cancer-associated alterations in cellular phenotypes within PBMCs. The main findings are summarized in Table 1.

**Table 1:** Association between blood cell phenotypes and different types of cancer.

Cancer	Cell Phenotype	References
CLL	CD5^+^CD19^+^CD23^+^ cells and NLCs derived from PBMC monocytes	[27,28]
cHL	Exhausted (increased GITR^+^, CD366^+^, CD152^+^, and/or PD1^+^), activated (CD272^+^), and differentiated (reduced CD127) T cells; increased Th17 and Tc17 cells	[29]
Melanoma	FKBP51s^+^ T lymphocytes	[9]
Melanoma	FKBP51s^+^ monocytes with immunosuppressive phenotype	[9]
CRC	CD177^+^ Tregs with increased activation potential	[30]
NSCLC	CD8^+^PD-1^high^ T lymphocytes with an exhausted phenotype	[24]
NSCLC	CD3^+^CTLA-4^+^ cells and Tregs (CD4^+^CD25^+^FOXP3^+^)	[11]
NSCLC	Tph (CD4^+^PD-1^+^CXCR5^−^) cells with heightened activation	[31]
HCC	Vδ1^+^ CD69^+^ γδ T cells with high cytotoxic potential	[32]
OSCC	TIGIT^+^ T cells with a dysfunctional phenotype (low proliferation and reduced cytokine secretion)	[18]
OSCC	Increased CD4^+^ T cells, phenotypic shift from naïve to memory/effector cells, higher frequency of exhausted phenotypes (PD-1^+^ and TIM-3^+^), increased Tregs, Th17 and Tc17 cells	[33]
Cervical cancer	PD-1^+^, TIGIT^+^ and/or Tim-3^+^ T and NK cells, including a NKG2D^+^ and DNAM-1^+^ subgroup	[17]
Breast cancer	MMP11^+^ cells	[34]
Endometrial cancer	Tregs with increased CD25 and FoxP3 expression	[35]

Note: CLL: chronic lymphocytic leukemia, NLCs: nurse-like cells, PBMCs: peripheral blood mononuclear cells, cHL: classical Hodgkin lymphoma, CRC: colorectal cancer, PD-1: programmed cell death protein 1, Th cells: helper T cells, Tc cells: cytotoxic T cells, Tregs: regulatory T cells, NSCLC: non-small cell lung carcinoma, Tph cells: peripheral helper T cells, HCC: hepatocellular carcinoma, OSCC: oral squamous cell carcinoma, NK cells: natural killer cells, NKG2D: NK group 2 member D receptor, DNAM-1: DNAX accessory molecule-1.

### 3.1 Chronic Lymphocytic Leukemia

Chronic lymphocytic leukemia (CLL) is characterized by the accumulation of mature CD5^+^CD19^+^CD23^+^ B cells in the peripheral blood [28]. In addition, other cells present in the TME, known as nurse-like cells (NLCs), play a fundamental role in supporting CLL cell survival and proliferation. These cells originate from CD14^+^ monocytes derived from PBMCs and are characterized by the expression of CD68, CD163, and stromal cell-derived factor 1 (SDF-1), among other markers. NLCs have been equated to tumor-associated macrophages and have been observed to be in close contact with CLL cells within the lymphoid tissues of patients [27].

### 3.2 Classical Hodgkin Lymphoma

In patients with classical Hodgkin lymphoma (cHL), immunological alterations have been identified that suggest phenotypes with a higher degree of exhaustion (increased frequencies of GITR^+^, CD366^+^, CD152^+^, and/or PD1^+^ subsets), activation (CD272^+^ cells), and differentiation (reduced CD127) of peripheral T cells in newly diagnosed patients compared to healthy individuals. In addition, a decrease in early T cells producing interferon-γ (IFN-γ) and tumor necrosis factor (TNF) has also been reported, with many of these alterations persisting after therapy. These extensive and systemic changes in T-cell subsets include shifts in their functional polarization, with a higher proportion of Th17 and Tc17 cells compared to Th1 cells. The loss of IFN-γ^+^ cells in cHL patients may remove physiological brakes on interleukin-17 (IL-17)-mediated responses. These findings provide possible explanations for the underlying causes of immune dysfunction observed in cHL and suggest that, in addition to PD-1, other immune checkpoints may represent relevant therapeutic targets for cHL treatment, such as CTLA-4, TIM-3, inducible T-cell costimulator (ICOS), B and T-lymphocyte attenuator (BTLA, CD272), TIGIT, glucocorticoid-induced tumor necrosis factor related (GITR), or the chemokine receptors CC-chemokine receptor 4 (CCR4) and CC-chemokine receptor 6 (CCR6), which are associated with Th17 cells [29].

### 3.3 Melanoma

FKBP51s is an isoform of the FKBP51 protein that plays a relevant role in tumor-related immunosuppression [36]. This protein is abundantly expressed in immune cells and is induced in response to coinhibitory immune receptor signaling. Immunophenotyping analyses of PBMCs from melanoma patients have shown an increase in T-lymphocyte and monocyte subsets, with a significantly higher proportion of FKBP51s^+^ cells compared with healthy controls [9].

### 3.4 Colorectal Cancer

Regulatory T cells (Tregs) mediate immunosuppression and are crucial for maintaining immune balance and tissue homeostasis [37]. In colorectal cancer (CRC), an increased frequency of CD177^+^ Treg cells has been reported, and these cells are more prone to activation. This increase correlates with a reduced antitumor response, thereby facilitating tumor immune evasion. In addition, CD177 promotes the transendothelial migration of both Tregs and CD8^+^ T cells, a property that could potentially be exploited to improve therapies based on Tregs or chimeric antigen receptor T cells (CAR-T) approaches [30,38,39].

### 3.5 Lung Cancer

Analysis of peripheral blood samples from patients with non-small cell lung carcinoma (NSCLC) has confirmed the presence of CD8^+^PD-1^high^ T lymphocytes with an exhausted phenotype prior to immunotherapy treatment [24]. Moreover, another study detected higher frequencies of CD3^+^CTLA-4^+^ cells and Treg cells (CD4^+^CD25^+^FOXP3^+^), together with elevated serum levels of interleukin-6 (IL-6) and transforming growth factor-β (TGF-β) [11].

Peripheral helper T (Tph) cells represent another T cell subset, characterized by a CD4^+^PD-1^+^CXCR5^−^ phenotype. These cells stimulate B cell responses in peripheral tissues and play an important role in inflamed tissues [40]. In patients with NSCLC, particularly lung adenocarcinoma, significant increase in the percentage of circulating Tph cells has been observed. Moreover, Tph cells exhibit heightened activation, as evidenced by increased ICOS expression, which has been associated with the invasive capacity of this cancer. These findings suggest that Tph cells may represent a promising therapeutic target in lung adenocarcinoma [31].

### 3.6 Hepatocellular Carcinoma

Gamma delta (γδ) T cells constitute a unique subset of T lymphocytes with innate-like features, due to their major histocompatibility complex (MHC)-unrestricted ability to activate and respond to stimuli. Among these, Vδ1 T cells represent the main γδ T cell population in the human liver. This subset includes both tissue-resident and recirculating cells that play a central role in maintaining hepatic immune homeostasis [41]. In hepatocellular carcinoma (HCC), Vδ1^+^CD69^+^ γδ T cells, which are difficult to access within the TME, have also been observedin the PBMCs of patients. These circulating cells exhibit higher cytotoxic potential and enhanced tumor reactivity compared with those from healthy donors. In this context, circulating Vδ1^+^CD69^+^ γδ T lymphocytes represent a promising candidate for the development of immunotherapeutic strategies targeting HCC [32].

### 3.7 Oral Squamous Cell Carcinoma

TIGIT has emerged as a promising therapeutic target in cancer immunotherapy and is highly expressed on several immune cell types, including activated T lymphocytes, NK cells, and Treg cells. Accumulating evidence demonstrates that TIGIT signaling modulates both T cell- and NK cell-mediated tumor recognition *in vitro* and *in vivo* [42]. In oral squamous cell carcinoma (OSCC), TIGIT is highly expressed on T cells derived from PBMCs, which display a dysfunctional phenotype, characterized by reduced proliferative capacity and decreased secretion of IL-2, TNF-α, and IFN-γ. Moreover, CD4^+^TIGIT^+^ T cells exhibit immunosuppressive properties, as evidenced by high Foxp3 expression and increased production of interleukin-1 (IL-1). Importantly, TIGIT blockade can improve *in vitro* both the proliferative capacity and cytokine production (IL-2, TNF-α, and IFN-γ) of CD4^+^ and CD8^+^ T cells from OSCC patients [18]. However, there are no *in vivo* studies demonstrating that TIGIT blockade restores cytokine production, and therefore its translational relevance has not yet been fully established in clinical settings.

Another study has also identified a series of distinctive features in T lymphocytes from OSCC patients, including an increased proportion of CD4^+^ T cells, a phenotypic shift from naïve to memory/effector subsets, higher frequency of exhausted T cells (PD-1 and Tim-3 expression, including Tregs), and an enrichment of Th17 and Tc17 cells. Imbalances in the Th17/Tc17 and Th17/Treg ratios have also been observed. These findings suggest that T lymphocytes contained in PBMCs may be involved in the development and progression of OSCC, representing a manifestation of the host immune response against tumor neoantigens [33].

### 3.8 Cervical Cancer

In women with cervical cancer and premalignant lesions, the percentages of T and NK cells expressing TIGIT, TIM-3, and PD-1 are increased in peripheral blood. These PD-1^+^ cells with an exhausted phenotype frequently coexpress TIGIT and/or TIM-3. Additionally, a subgroup of these cells expresses NK group 2 member D receptor (NKG2D) and DNAX accessory molecule-1 (DNAM-1). These findings provide an overview of the immune response status against precancerous lesions and cervical cancer and may offer an early indication of which patients could benefit from immune checkpoint inhibitor therapies [17].

### 3.9 Breast Cancer

Matrix metalloproteinases (MMPs) are zinc-dependent metalloproteolytic enzymes that contribute to tumor progression through extracellular matrix (ECM) degradation and by promoting the release of cytokines, growth factors, and cell surface-associated molecules. Among them, matrix metalloproteinase-11 (MMP11) plays a key role as it is produced by cancer cells, stromal cells, and cells within the surrounding TME [43]. In PBMCs from breast cancer patients, a subpopulation (25.9%) exhibiting high MMP11 gene expression has been identified. It has been observed that MMPs expression in PBMCs is regulated by the microenvironment, whereas the expression of inflammatory genes in normal fibroblasts or cancer-associated fibroblasts is differentially regulated by PBMCs. These findings highlight the importance of stromal cell communication and suggest that PBMCs play a role in promoting aggressive tumor behavior [34].

### 3.10 Endometrial Cancer

The presence of endometrial cancer cells has been found to induce phenotypic changes in PBMCs of endometrial cancer patients. Specifically, Treg cells exhibit significantly increased expression of CD25 and FoxP3, reflecting an enhanced immunosuppressive profile [35].

## 4 Association between Cellular Phenotypes and Disease Stage

Several studies have associated PBMC cellular phenotypes in cancer patients with disease progression and specific clinical stages. A summary of the available data is provided in Table 2.

**Table 2:** Association of blood cell phenotypes with disease stage and progression.

Cancer	Disease Stage/Progression	Cellular Phenotypes	References
CRC	Secondary PC	More naïve CD8^+^ T cells profile	[14]
NSCLC	Increasing degree of tumor invasion	Positive correlation with Tph cells	[31]
HCC	Smaller tumor size and improved clinical prognosis	Increased frequencies of Vδ1^+^CD69^+^ γδ T cells	[32]
OSCC	Advanced T stage and lymph node invasion	High TIGIT expression in CD4^+^ and CD8^+^ T cells	[18]
OSCC	Larger tumor size	Increased proportion of CD4^+^ T cells	[33]
OSCC	Advanced clinical stage and lymph node metastasis	Increased frequency of memory/effector and exhausted T-cell phenotypes	[33]
OSCC	Early clinical stage and absence of lymph node metastasis	Higher Th17/Treg ratio	[33]
Cervical cancer	Advanced disease stage	Slight increase in total NK cells (reduced CD56^bright^ and increased CD56^dim^ subsets), loss of NKp30^+^ NK cells, reduced perforin levels, increased soluble B7H6	[44]
Breast cancer	HER2-negative metastatic disease	Reduced CD4^+^ T lymphocytes and plasmacytoid dendritic cells; increased monocytes and Tregs; altered effector T cells, Tregs, and B-cells phenotypes; increased expression of activation and exhaustion markers (PD-1, TIGIT, TIM-3, ICOS), shift toward a Th2/Th17 profile	[15]

Note: CRC: colorectal cancer, PC: peritoneal carcinomatosis, NSCLC: non-small cell lung carcinoma, Tph cells: peripheral helper T cells, HCC: hepatocellular carcinoma, GC: gastric cancer, TNM: tumor-node-metastasis, GCSCs: gastric cancer stem cells, OSCC: oral squamous cell carcinoma, TIGIT: T-cell immunoreceptor with Ig and ITIM domains, NK cells: natural killer cells, Th cells: helper T cells, Tregs: regulatory T cells.

### 4.1 Colorectal Cancer

In CRC patients with secondary peritoneal carcinomatosis (PC), a more naïve profile of CD8^+^ T cells has been observed in peripheral blood and intra-abdominal adipose tissue compared with CRC patients presenting advanced disease but without PC [14]. This finding suggests that distinct patterns of systemic immune modulation may be associated with peritoneal dissemination.

### 4.2 Lung Cancer

In NSCLC patients, particularly lung adenocarcinoma, increased numbers of Tph cells exhibiting heightened immune activity have been detected in both circulation and in tumor tissue, correlating positively with the degree of tumor invasion. These findings suggest that the TME may promote Tph cells accumulation as a mechanism contributing to tumor progression and malignancy [31].

### 4.3 Hepatocellular Carcinoma

In HCC, an increase in the Vδ1^+^CD69^+^ γδ T cells has been associated with smaller tumor size and improved clinical prognosis. These cells, present in PBMCs, exhibit enhanced cytotoxicity. HCC patients with high simultaneous expression of TRDV1 and CD69 show increased production of effector molecules and prolonged overall survival [32]. Together, these observations support a protective role for circulating Vδ1^+^CD69^+^ γδ T cells in HCC progression. These findings highlight the functional heterogeneity of γδ T cells, suggesting that their prognostic impact may depend on activation state, tissue trafficking, and tumor context.

### 4.4 Oral Squamous Cell Carcinoma

In the PBMCs of OSCC patients, high TIGIT expression on CD4^+^ and CD8^+^ T cells has been associated with advanced T stage and lymph node invasion [18]. The proportion of CD4^+^ T cells is significantly increased in patients with larger tumors compared with those bearing smaller tumors. Memory/effector and exhausted T cell phenotypes are significantly associated with advanced clinical stage and lymph node metastasis, whereas a higher Th17/Treg ratio correlates with early clinical stages and absence of lymph node metastasis [33]. These findings suggest that systemic T cell phenotypes reflect disease progression in OSCC.

### 4.5 Cervical Cancer

NK cells play a central role in tumor immunosurveillance through a delicate balance between activating and inhibitory receptors, as well as through the release of cytokines and chemokines [45]. In patients with cervical cancer and precursor lesions, an altered immune phenotype has been identified that correlates with disease progression from cervical intraepithelial neoplasia grade 1 (CIN 1) to International Federation of Gynecology and Obstetrics (FIGO) stage IV. This abnormal immune profile is observed in peripheral NK cells and exhibits characteristics typically associated with aged NK cells [44]. Disease progression is associated with a slight increase in total NK cell numbers and a redistribution of their subsets, characterized by a decrease in CD56^bright^ cells and an increase in CD56^dim^ cells. In addition, a loss of NKp30^+^ NK cells, particularly the NKp30C isoform, has been reported, along with decreased perforin levels and overexpression of soluble B7H6, the ligand for NKp30. This dysfunctional phenotype appears to be influenced, at least in part, by the presence of tumor cells, possibly due to direct contact with B7H6 [44]. Natural cytotoxic receptors (NKp30, NKp46, and NKp44) constitute an important group of activation receptors that recognize cellular stress molecules. Among them, NKp30 plays a key role in initiating NK cell-mediated cytotoxic response and has been linked with the lysis of tumor cells [46]. Future therapeutic strategies should aim to restore NK cell fitness by enhancing the expression of NKp30 isoforms and restoring perforin levels. Overall, these findings describe a state of immune dysfunction in cervical cancer, highlighting the relevance of NK cell alterations for the development of novel immunotherapeutic approaches [44].

### 4.6 Breast Cancer

Patients with HER2-negative metastatic breast cancer exhibit significantly increased levels of circulating monocytes, together with reduced levels of CD4^+^ T lymphocytes and plasmacytoid dendritic cells. These patients also display elevated numbers of Treg cells and altered phenotypes of effector T cells, Tregs, and B cells. Higher expression of immune checkpoints such as PD-1, TIGIT, TIM-3, and ICOS has also been observed, along with other activation and exhaustion markers, as well as a shift toward Th2 and Th17 polarization [15]. 

The identified T-cell phenotypes correlate with functionality, as assessed by IFN-γ production. Moreover, a subset of CD4^+^ T cells coexpressing multiple immune checkpoint receptors has been detected and is negatively associated with intratumoral CD4^+^ T-cell infiltration. In conclusion, the systemic immune signatures identified reflect an immunosuppressed environment in patients with metastatic breast cancer who have progressed or relapsed after standard treatments, and are consistent with a state of persistent chronic inflammation. These activated immunosuppressive mechanisms may be explored as potential therapeutic targets, as well as biomarkers of treatment response or resistance [15].

## 5 Association of Cellular Phenotypes with Treatment Response

A growing number of studies have associated PBMC phenotypes in cancer patients with therapeutic response and clinical outcome. The main observations are summarized in Table 3.

**Table 3:** Association between cancer treatments and circulating blood cell phenotypes.

Cancer	Treatment	Associated Blood Cell Phenotypes	References
CLL	AHCC	Reduced number of NLCs and phenotypic alterations	[27]
cHL	ABVD chemotherapy	Increased frequencies of HLA-DR^+^, CD95^+^ and TIGIT^+^ phenotypes	[29]
Melanoma	Anti-PD-1	Responders: increased FKBP51s^+^ Tregs	[9]
Melanoma	Anti-PD-1	Non-responders: early increase in FKBP51s^+^PD-L1^+^ monocytes	[9]
Melanoma	Mocetinostat + nivolumab + ipilimumab	Reduction in MDSCs and anti-inflammatory monocyte phenotypes	[47]
Metastatic CRC	TARE of liver metastases	Increased CD3^+^ T cells and CD8^+^ Ki-67^+^ cells, reduced CD4^+^ CTLA-4^+^ T cells	[48]
Lung cancer	CT/IT	Increased Lin-CD34^+^DNAM-1^bright^ and CD34^+^DNAM-1^bright^CXCR4^+^ cells	[49]
NSCLC	Pembrolizumab or atezolizumab	Higher CD5 expression on Tem cells associated with reduced CD8^+^ T-cell differentiation and improved clinical outcomes	[50]
NSCLC	Atezolizumab	Treatment efficacy correlated with exhausted T-cell phenotypes and increased TCR repertoire diversity	[24]
NSCLC (stage IA-IIIA)	Lobectomy	Transient increase in M-MDSCs and reduction in CD3^+^ T cells, CD8^+^ T cells and Tem cells	[51]
HCC	Anti-PD-1	Non-responders: increased frequency of CD14^+^GSK3β^+^ cells	[5]
HCC	RFA	Increased monocytes; reduced CD8^+^ Tem cells, T-cell activation and cytotoxicity	[12]
OSCC (stage III/IV)	Nivolumab (neoadjuvant)	MPR: increased CD8^+^ T-cell activation (higher PD-1, TIGIT, and IFN-γ expression, and reduced PD-L1 expression)	[52]
HER2^+^ breast cancer	Chemotherapy + trastuzumab and/or lapatinib	Increased CD4^+^ and CD8^+^ T cells; reduced NK cells, B cells and monocytes; decreased cytotoxicity	[53]
Esophageal cancer	Atezolizumab + nCRT + esophagectomy	Incomplete response: increased Tregs and intermediate monocytes, reduced cDC2, elevated immunosuppressive cytokines	[13]
Esophageal cancer	Atezolizumab + nCRT + esophagectomy	Post-surgery: activated CD40^+^ monocytes; low CD8^+^Ki67^+^ T-cell frequencies; enrichment of CD206^+^ monocytes associated with early recurrence	[13]
Metastatic prostate cancer	Ipilimumab	Responders: expansion of Tem cell, increased T-bet expression in T cells	[54]
Advanced rare cancers (dMMR/MSI-H)	Nivolumab	Responders: enrichment of T-bet^+^PD-1^+^CD4^+^ cells	[55]

Note: CLL: chronic lymphocytic leukemia; AHCC: active hexose correlated compound, NLCs: nurse-like cells, cHL: classical Hodgkin lymphoma, ABVD: adriamycin, bleomycin, vinblastine, dacarbazine; Tregs: regulatory T cells, PD-L1: programmed death-ligand 1, MDSCs: myeloid-derived suppressor cells, CRC: colorectal cancer, TARE: transarterial radioembolization, CT/IT: chemotherapy plus immunotherapy, DNAM-1: DNAX accessory molecule-1, NSCLC: non-small cell lung carcinoma, Tem cells: effector memory T cells, TCR: T-cell receptor, M-MDSC: monocytic myeloid-derived suppressor cells, HCC: hepatocellular carcinoma, RFA: radiofrequency ablation, OSCC: oral squamous cell carcinoma, PD-1: programmed cell death protein 1, IFN-γ: interferon-γ, NK cells: natural killer cells, MPR: major pathological response, nCRT: neoadjuvant chemoradiotherapy, cDC2: conventional type 2 dendritic cells, dMMR: deficient mismatch repair, MSI-H: microsatellite instability-high.

### 5.1 Chronic Lymphocytic Leukemia

Active hexose correlated compound (AHCC) is a Basidiomycotina-derived extract used as nutritional supplement that is generally well tolerated, with no reported adverse effects. It exhibits potential antitumor and immunomodulatory activity, acting on monocytes, NK cells, T cells, and natural killer T (NKT) cells [27,56]. In PBMCs from patients with CLL, *in vitro* treatment with AHCC significantly reduces the number of NLCs and alters their phenotype. This reduction is remarkable and statistically significant at concentrations of 5 and 10 mg/mL, suggesting that AHCC antagonizes NLC differentiation [27].

### 5.2 Classical Hodgkin Lymphoma

Combination chemotherapy with doxorubicin, bleomycin, vinblastine and dacarbazine (ABVD) is the standard treatment for advanced Hodgkin lymphoma, achieving failure-free survival rates of 60%–70% and overall survival rates of 80%–90% [57]. In cHL patients, cell subsets expressing combinations of PD-1^+^ and CD366^+^ are elevated prior to treatment, while those expressing HLA-DR^+^, CD95^+^, or TIGIT^+^ are increased after ABVD chemotherapy. The results suggest that circulating exhausted cells present before treatment are replaced by activated cells (HLA-DR^+^), with a persistent population of TIGIT^+^ exhausted cells remaining after chemotherapy. Moreover, approximately three months post-treatment, patients continue to exhibit alterations in T-cell immune checkpoints and functional profiles [29]. Longitudinal data beyond three months post-treatment are currently limited, and further studies are needed to assess whether these immune alterations persist or act as resistance biomarkers.

### 5.3 Melanoma

The protein FKBP51s marks a subset of Tregs with high suppressive activity that has been associated with improved responses to ICI therapies in melanoma. In patients treated with anti-PD-1 antibody, an increase in circulating FKBP51s^+^ Treg cells was observed in responders and correlated with improved prognosis. It should be noted that isoform-specific validation of FKBP51s is still lacking. However, significant changes in this population occurred only several weeks after treatment initiation. In contrast, non-responders exhibited an early increase in FKBP51s^+^PD-L1^+^ monocytes during therapy. These findings suggest that FKBP51s expression may serve as a biomarker to guide patient selection and to monitor responses to ICI in melanoma [9]. Importantly, the delayed increase in FKBP51s^+^ Tregs observed in responders suggests that these changes may reflect treatment-induced immune modulation rather than predictive baseline biomarkers. In addition to lymphoid alterations, increased frequencies of FKBP51s^+^PD-L1^+^ circulating monocytes have also been reported in melanoma patients, suggesting that myeloid-mediated systemic immunosuppression may contribute to reduced responsiveness to anti-PD-1 therapy.

In a phase Ib clinical trial (NCT03565406) designed to evaluate the combination of mocetinostat with nivolumab (anti-PD-1) and ipilimumab (anti-CTLA-4) in previously untreated patients with unresectable stage III/IV metastatic melanoma, safety, tolerability, clinical efficacy, and immune correlates were assessed. Patient PBMCs showed significant reductions in myeloid-derived suppressor cells (MDSCs), along with a trend toward decreased anti-inflammatory monocyte phenotypes. Immune correlates supported a shift from immunosuppressive toward effector immune profiles, indicating that mocetinostat may enhance antitumor immunity by reprogramming both T lymphocytes and myeloid cells [47].

### 5.4 Colorectal Cancer

In patients with metastatic CRC following yttrium-90 transarterial radioembolization (TARE) of liver metastases, longitudinal immune monitoring revealed significant treatment-induced changes in circulating immune cells. An increase in CD3^+^ T cells and a decrease in CD4^+^ CTLA-4^+^ T cells were observed three weeks after treatment, together with elevated proportions of proliferating CD8^+^ Ki-67^+^ T cells, at three and six weeks after treatment. These changes were accompanied by increased number of circulating antigen-presenting cells three weeks after TARE, coinciding with enhanced IFN-γ production, and reduced levels of interleukin-10 (IL-10). Collectively, these findings show an immunological shift from a protumoral profile toward an antitumoral profile, favoring a Th1/CD8^+^ T-cell response, and support the rationale for combining TARE with immunotherapeutic strategies in metastatic CRC [48].

### 5.5 Lung Cancer

In patients with advanced lung cancer, PBMC samples were analyzed immediately before treatment initiation and prior to the second cycle of concurrent chemotherapy plus immunotherapy (CT/IT) with pembrolizumab (anti-PD-1), durvalumab (anti-PD-L1), and atezolizumab (anti-PD-L1). The study revealed a five-fold increase in the frequency of Lin^−^CD34^+^DNAM-1^bright^ cells following therapy. Moreover, inflammatory precursors characterized as CD34^+^DNAM-1^bright^CXCR4^+^ also expanded and migrated into affected tissues, where they generated functional progenies. These findings suggest that this subset may play a relevant role in maintaining immune balance within the TME, which is strongly influenced by the SDF-1/CXCR4 axis in a pro-metastatic context [49].

CD5 plays an important role in the immune regulatory network which controls autoimmune processes and helps protect against the development of autoimmunity [58]. In patients with NSCLC, higher CD5 expression on effector memory T cells (Tem) was associated with a less differentiated CD8^+^ T cells phenotype and improved clinical outcomes in response to therapy with pembrolizumab or atezolizumab. These findings highlight CD5 expression as a dynamic marker of CD8^+^ T cell differentiation, carrying important implications for the development of predictive biomarkers of response to ICI therapy [50].

T cells responses are driven by unique T-cell receptors (TCRs) that specifically recognize antigens from a variety of biological contexts. Consequently, analyzing the T-cell repertoire provides valuable insights into immune responses and into the mechanisms underlying diseases such as cancer [59]. In patients with advanced NSCLC treated with atezolizumab, analysis of PBMCs confirmed that the presence of exhausted T cells and the diversity of the TCR repertoire correlate with treatment efficacy. Therefore, monitoring the diversity index or the reshaped TCR repertoire patterns induced by effective PD-L1 blockade could serve as useful biomarkers during immunotherapy [24].

The impact of surgical stress on PBMC phenotypes has also been investigated in patients with stage IA-IIIA NSCLC undergoing lobectomy. Surgery induced a transient reduction in total CD3^+^ T cells, CD8^+^ T cells, and Tem cells, along with an increase in monocytic-MDSCs (M-MDSCs). These alterations resolved within four weeks after the procedure and were comparable between patients undergoing open thoracotomy or minimally invasive video-assisted thoracoscopic surgery (VATS), indicating that surgical approach does not differentially affect postoperative immune recovery [51].

### 5.6 Hepatocellular Carcinoma

Glycogen synthase kinase-3 (GSK-3), particularly the GSK-β isoform, is a conserved serine/threonine kinase implicated in tumor initiation and progression [60]. In patients with HCC treated with anti-PD-1 therapy, non-responders exhibited a higher percentage of circulating CD14^+^GSK3β^+^ cells compared with responders. Therefore, the presence of these cells may non-invasively predict sensitivity to anti-PD-1 treatment. This finding provides new strategies to anticipate the response to anti-PD-1 immunotherapy, enhance its therapeutic effect, and bring new hope to patients with HCC [5].

Radiofrequency ablation (RFA), a curative treatment for non-surgical HCC patients, has also been shown to induce systemic immune alterations. In patients with recurrent HCC undergoing repeated RFA, a significant increase in the proportion of monocytes and a concomitant decrease in multiple T-cell subsets were observed shortly after treatment. The results indicated that RFA therapy may enhance the antigen-presenting capacity of monocytes; however, this effect is insufficient to induce a robust antitumor immune response. This is because RFA also appears to exert an immunosuppressive effect by reducing the T-cell population and activation, decreasing the expression of key markers such as CD161 and CD5, reducing the expression of genes involved in T-cell cytotoxic function (GZMB, GZMH, GZMK, CD8A) and decrease in effector and memory CD8^+^ T cells. In addition, tumor-derived components promoted vascular endothelial growth factor (VEGF) secretion by monocytes, Treg cells, B cells, and naïve CD4^+^ T cells. These findings suggest that combining RFA with immunoenhancing strategies could represent a promising therapeutic approach in patients with recurrent HCC [12].

### 5.7 Oral Squamous Cell Carcinoma

In patients with stage III/IV OSCC treated with neoadjuvant nivolumab prior to surgery, PBMC immunophenotyping revealed higher levels of CD8^+^ T-cell activation in patients achieving a major pathological response (MPR). This activation was characterized by increased expression of PD-1, TIGIT, and IFN-γ, along with lower PD-L1 levels. These results support that neoadjuvant nivolumab treatment in advanced-stage oral cancers is safe and capable of inducing MPR, with treatment efficacy associated with the activation status of peripheral T-lymphocyte populations [52].

### 5.8 Breast Cancer

About 20% of breast cancers overexpress human epidermal growth factor receptor 2 (HER2), which is associated with aggressive tumor behavior, increased proliferation, invasiveness, angiogenesis, and metastasis, leading to poorer prognosis and reduced survival [61]. The phase II neoadjuvant clinical trial ICORG10-05 (NCT01485926) compared chemotherapy combined with trastuzumab (anti-HER2), lapatinib, or both in patients with HER2-positive breast cancer. The therapy reduced the cytotoxic activity of circulating immune cells, associated with increases in CD4^+^ and CD8^+^ T cells, along with decreases in NK cells, monocytes, and B cells. Significant immune cell changes were predominantly observed in patients with residual disease who did not achieve a pathological complete response. Overall, PBMCs exhibited altered phenotype and functionality after completion of neoadjuvant treatment [53].

### 5.9 Esophageal Cancer

A translational substudy of the PERFECT trial (NCT03087864) investigated immune correlates of response to atezolizumab in combination with neoadjuvant chemoradiotherapy (nCRT) followed by esophagectomy in patients with resectable esophageal adenocarcinoma. Peripheral blood analysis revealed distinct systemic immune profiles between patients with complete and incomplete responses. Incomplete responders exhibited increased immunosuppressive subsets, including Treg cells and intermediate monocytes, reduced frequencies of conventional type 2 dendritic cells (cDC2) and elevated immunosuppressive cytokines. At baseline, these immunosuppressive phenotypes correlated with tumor gene expression signatures associated with the Wnt/β-catenin pathway, while on-treatment signatures were linked to epithelial-mesenchymal transition and angiogenesis. After surgery, early recurrence was associated with CD40^+^ monocytes activation, low frequencies of CD8^+^Ki67^+^ T cells, and enrichment of CD206^+^ monocytes. The enrichment of CD206^+^ monocytes further supports the contribution of immunosuppressive myeloid populations to tumor recurrence and impaired response to immunochemoradiotherapy. This study identified systemic immunosuppressive barriers to neoadjuvant immunochemoradiotherapy and highlighted potential therapeutic targets for future clinical trials [13].

### 5.10 Prostate Cancer

In patients with metastatic prostate cancer exhibiting an incomplete biochemical response to initial androgen deprivation therapy, treatment with ipilimumab, an anti-CTLA-4 antibody, was associated with expansion of Tem cell subsets and increased T-bet expression in T cells. These changes suggest the induction of a Th1-type immune response and were associated with clinical benefit [54].

### 5.11 Rare Advanced Cancers

Deficient mismatch repair (dMMR) and high microsatellite instability (MSI-H) are established predictive biomarkers for ICIs therapy. In a multicenter phase II clinical trial evaluating nivolumab monotherapy in patients with rare advanced dMMR/MSI-H cancers, immune phenotyping revealed a specific enrichment of T-bet^+^PD-1^+^CD4^+^ lymphocytes in responders. This subset, previously suggested to be tumor-reactive, was associated with favorable clinical outcomes. Although validation in larger cohorts is required, the proportion of circulating T-bet^+^PD-1^+^CD4^+^ cells may represent a novel predictive biomarker for response to nivolumab in rare cancers [55].

## 6 Clinical Relevance and Future Directions of PBMC Immunophenotyping in Cancer

Immune cells express a wide variety of proteins, and the assessment of their different combinations enables accurate identification of the cellular subsets involved in disease processes. Immune responses are coordinated by a complex network of proteins distributed across multiple cell types. Thus, in the context of malignant neoplasms, the presence or absence of specific cell types can significantly influence the effectiveness of the antitumor immune responses [29].

Emerging evidence indicates that peripheral immune cells do not merely reflect systemic immune status. They also actively participate in bidirectional communication with the TME. Tumor-derived factors, including cytokines, chemokines, and extracellular vesicles such as exosomes, may systemically reprogram circulating immune cells toward immunosuppressive or dysfunctional states. Overall, exosomes released by tumors carry molecules that enhance cancer cell proliferation, invasion, and the development of drug resistance. Consequently, these tumor-derived exosomes have a crucial role in mediating communication between cancer cells and the TME, which consists of stromal components, immune cells, and the extracellular matrix. Conversely, PBMC subsets may contribute to tumor progression by secreting soluble mediators. These factors modulate stromal remodeling, angiogenesis, and immune escape [62]. These interactions further support the relevance of PBMC phenotypes as systemic readouts of tumor-driven immune modulation.

The establishment of adaptive immune responses against cancer involves not only the tumor tissue but also the peripheral blood [33]. Therefore, analyzing the expression of immune checkpoint receptors in the PBMCs of cancer patients, together with evaluating how blood immune phenotypes correlate with T cell functional responses, is of considerable clinical relevance [15]. Furthermore, increased infiltration of myeloid cells within the TME, such as immune suppressive monocytic and granulocytic MDSCs, is associated with poor prognosis, reduced survival, and resistance to therapy. These cells promote an immunosuppressive environment by inhibiting T and NK cell activity through immune checkpoint ligand expression, including PD-L1 and TIM-3, and secretion of suppressive cytokines (e.g., IL-10, TGF-β). They support tumor progression by enhancing proliferation, angiogenesis, extracellular matrix remodeling, and metastatic dissemination. Overall, their presence correlates with advanced disease stages and unfavorable clinical outcomes [16,63].

Although several studies have reported associations between circulating cell phenotypes and different cancer types (CLL [27], HCC [29], melanoma [9], CRC [30], NSCLC [11,24,31], HCC [32], HNSCC [18,33], cervical cancer [17], breast cancer [34], and endometrial cancer [35]), there remains a clear scarcity of research in this field and, consequently, a limited amount of available data to establish precise phenotypic patterns that allow the characterization of different cancer types. Despite this limitation and the variability across tumor types, there is a consistent trend for PBMCs from cancer patients to exhibit highly activated or activation-prone phenotypes, as well as features of immune exhaustion [29,30,31,33]. These exhausted effector cells, that is, putatively cytotoxic lymphocytes that have lost their ability to effectively participate in antitumor responses, are key to advancing biomolecular and clinical discovery [13].

Among the predominant cell populations associated with cancer are Treg, Th17, and Tph cells [11,29,30,31,33,35], whose roles in cancer are still under investigation. Treg cells are well recognized for their potent immunosuppressive functions and their essential role in maintaining immune homeostasis. Within the TME, they suppress antitumor responses through the secretion of cytokines such as TGF-β, IL-10, and IL-35, as well as through the expression of surface molecules including CTLA-4 and PD-L1. Increased Treg frequencies have been consistently correlated with a reduction in antitumor immune responses, thereby facilitating tumor immune evasion [17,30,33].

The role of Th17 cells, which produce IL-17A, in carcinogenesis is controversial due to their plasticity and functions within TMEs. They exert antitumor immunity by promoting dendritic cells recruitment, enhancing cytotoxic activity, improving Th1 responses, and increasing MHC antigen expression. Conversely, they may also play a protumoral role by inducing tumor vascularization [33]. The involvement of Tph cells in cancer has also generated controversy in recent years. Initially described in inflammatory diseases, Tph cells have now been implicated in the pathogenesis of a wide range of conditions, including certain malignancies, although their precise function within tumors remains to be fully elucidated [31].

PBMC-derived myeloid populations have also been identified, and these cells can adopt immunosuppressive and tumor-promoting functions, thereby contributing to systemic immune dysregulation in cancer. Among them, nurse-like cells (NLCs) play a key role in the microenvironment of chronic lymphocytic leukemia (CLL), where they support tumor cell survival, proliferation, and drug resistance. These cells originate from the myeloid lineage and are driven by CLL cells to differentiate into a tumor-supportive phenotype. In this context, they are functionally comparable to tumor-associated macrophages and represent a relevant therapeutic target [27].

Several studies have also reported increased expression of immune checkpoint molecules such as CTLA-4, PD-1, TIM-3, and TIGIT in PBMCs from cancer patients [11,17,18,24,29,31,33]. These molecules have emerged as central targets in modern oncology, and their therapeutic modulation has established a new paradigm in cancer treatment through the development of ICIs [6,17,64,65,66].

Although most studies describe PBMC immunophenotypes in a cancer-specific context, a cross-cancer integrative perspective reveals the existence of conserved systemic immune signatures. Across multiple malignancies, PBMCs consistently exhibit features of T cell exhaustion, regulatory T cell expansion, and upregulation of immune checkpoint molecules such as PD-1, TIGIT, and TIM-3. These pan-cancer patterns suggest the presence of shared mechanisms of systemic immune dysregulation driven by chronic antigen exposure and tumor-derived immunomodulatory signals.

At the same time, cancer-type-specific phenotypic variations are also observed, including the expansion of Tph cells in NSCLC [31], Vδ1^+^CD69^+^ γδ T cells in HCC [32], or TIGIT^+^ dysfunctional T cells in OSCC [18]. These differences likely reflect tumor-specific microenvironmental cues, tissue tropism, and distinct inflammatory contexts, highlighting the need for context-dependent interpretation of PBMC biomarkers.

Importantly, these immune alterations are not merely descriptive but may contribute to therapeutic resistance through defined biological mechanisms. Expanded Treg populations and immunosuppressive myeloid cells, including monocytes and myeloid-derived suppressor cells (MDSCs), can inhibit effector T cell responses via cytokine secretion (e.g., IL-10, TGF-β) and metabolic regulation. In parallel, chronically stimulated CD8^+^ T cells progressively acquire an exhausted phenotype characterized by reduced cytotoxic function and sustained expression of inhibitory receptors. In addition, systemic immune alterations reflected in PBMCs are closely linked to tumor-immune crosstalk, including cytokine networks and chemokine-mediated trafficking (e.g., SDF-1/CXCR4 axis), which further shape treatment response and resistance.

Taken together, these findings support a model in which PBMC immunophenotypes reflect both shared and tumor-specific immune adaptations. These phenotypes integrate systemic and tumor-local signals. This dual perspective is essential for understanding their role as biomarkers and for the development of strategies aimed at overcoming resistance to cancer therapies.

**Figure 1:**
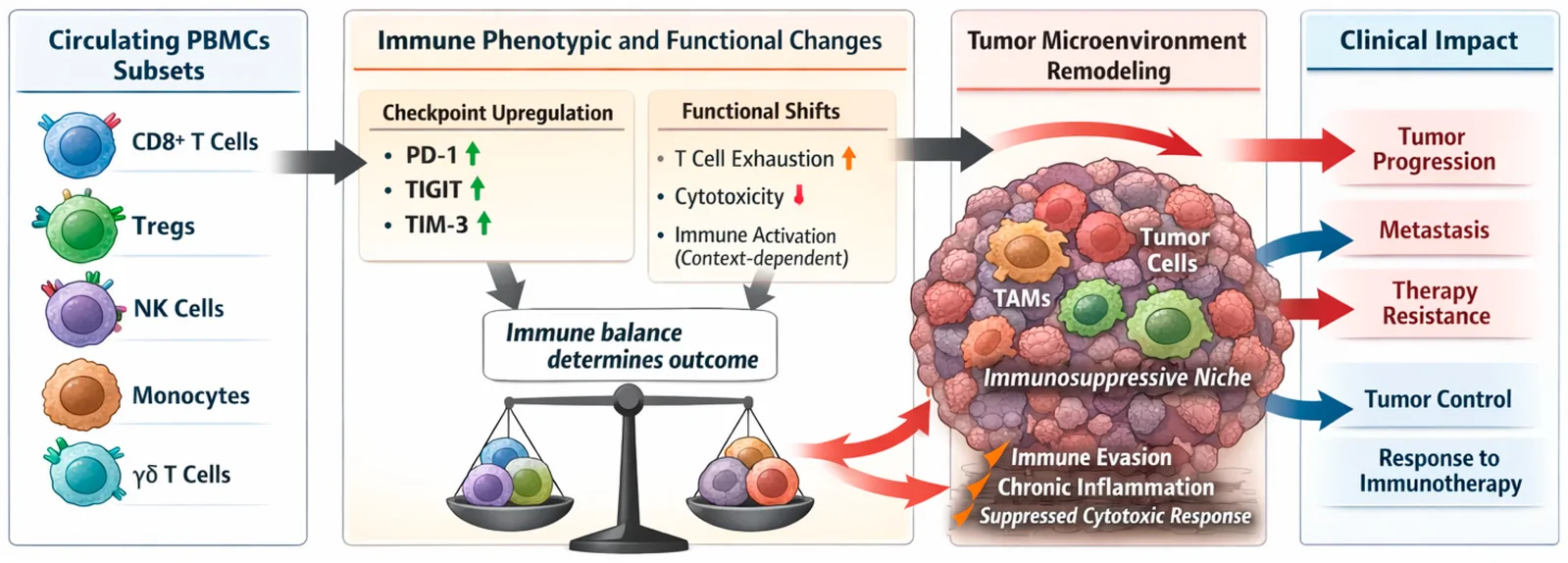
Integrative schematic linking PBMC alterations to tumor microenvironment remodeling and clinical outcomes. Circulating PBMC subsets undergo phenotypic and functional changes, including immune checkpoint upregulation and T-cell exhaustion. These alterations contribute to tumor microenvironment remodeling, promoting either immunosuppression and tumor progression or, alternatively, effective anti-tumor responses. The balance between these opposing immune forces ultimately determines clinical outcomes, including therapy response or resistance. The colored arrows indicate directionality of change: green upward arrows denote increased expression or upregulation, red downward arrows indicate decreased expression or downregulation, and orange arrows represent context-dependent functional shifts (e.g., T cell exhaustion and altered cytotoxic activity). PBMCs: peripheral blood mononuclear cells; CD: cluster of differentiation; Tregs: regulatory T cells; NK cells: natural killer cells; γδ T cells: gamma delta T cells; PD-1: programmed cell death protein 1; TIGIT: T-cell immunoreceptor with Ig and ITIM domains; TIM-3: T-cell immunoglobulin and mucin-domain containing-3; TAMs: tumor-associated macrophages.

To integrate the findings of this review, we generated a conceptual schematic illustrating the relationship between PBMC alterations, immune functional changes, tumor microenvironment remodeling, and clinical outcomes (Fig. 1). This model highlights how systemic immune dysregulation translates into local tumor-promoting or tumor-controlling mechanisms, ultimately influencing disease progression and therapeutic response.

Beyond these mechanistic insights, an important question is how these immune alterations translate into clinical outcomes. The poor prognosis observed in many cancers is driven by multiple factors, including diagnosis at advanced disease stages, tumor heterogeneity, and incomplete understanding of cancer biology [11]. This review provides an initial foundation for interpretation of cancer-associated immune phenotypes, which must be strengthened through a greater number of studies. Importantly, PBMC immunophenotypes not only differ across cancer types but also appear to correlate with treatment response and tumor progression, thereby acquiring prognostic and predictive relevance [9].

Studies analyzing the association between cellular phenotype and disease stage or progression are very limited. Existing reports mainly focus on the degree of tumor invasion, clinical prognosis, and patient survival, describing associations between distinct immune phenotypes and disease progression [14,15,18,31,32,33,44]. Moreover, the results are highly heterogeneous and do not allow the establishment of a clear predictive pattern of disease evolution, probably due to marked differences among tumor types, as well as their progression and aggressiveness. Therefore, increasing research efforts in this area is essential in order to identify patterns that may facilitate prognosis and, consequently, optimize therapeutic strategies.

In this context, the relationship between PBMC phenotypes and treatment response becomes particularly relevant. The limited response to conventional therapies justifies the need for new therapeutic approaches, particularly immunotherapeutic strategies such as immune checkpoint blockade and adoptive immune cell transfer. Several studies analyzing PBMCs from cancer patients undergoing different treatments have identified therapy-associated alterations in immune checkpoint molecules and immune cell composition [5,9,12,13,24,27,29,47,48,49,50,51,52,53,54,55]. As observed for disease progression, these results are highly heterogeneous, due not only to differences among different cancer types and clinical stages at which treatment effects were evaluated, but also to the wide variety of therapies employed. Nonetheless, systematic evaluation of immune checkpoint expression and function in circulating T cells, and their association with clinical outcomes, may provide valuable insights into treatment efficacy and resistance mechanisms.

The therapeutic interventions analyzed in these studies include natural compounds such as AHCC [27]; conventional treatments such as chemotherapy [13,29,49,53], TARE [48], lobectomy [51] and RFA [12]; as well as more recent immunotherapeutic strategies [5,9,13,24,47,49,50,52,53,54,55]. The predominance of immunotherapy-based or combinatorial approaches reflects the growing interest in harnessing immune mechanisms for cancer treatment. In general, immune checkpoint-targeted immunotherapies act by reactivating previously sensitized T cells against specific tumor antigens, thereby promoting tumor cell destruction. Despite their clinical success, these therapies are frequently associated with immune-related adverse events, especially in combination regimens, and a substantial proportion of patient fails to respond or develop resistance [9,67,68,69,70].

Among the immunotherapeutic agents investigated are antibodies targeting PD-1 [5,9,47,49,50,52,55] (e.g., nivolumab, pembrolizumab), CTLA-4 [47,54] (e.g., ipilimumab), PD-L1 [13,24,49,50] (e.g., atezolizumab, durvalumab), and HER2 (e.g., trastuzumab) [49]. Results vary widely across studies, as they differ in treatment, cancer type, stage, and clinical characteristics. However, some recurring immune features have been reported, such as increased post-treatment T-bet expression in prostate cancer and advanced rare cancers [54,55]. The TME can suppress antitumor immune responses, contributing to treatment resistance. ICIs help restore immune activity by reactivating exhausted T cells and promoting immune cell infiltration into tumors. This is particularly relevant in contexts where resistance is driven by immunosuppressive mechanisms such as increased regulatory T cells or myeloid-derived suppressor cells. Combining checkpoint inhibitors with other therapies, including immunotherapy, chemotherapy, or targeted treatments, has emerged as an effective strategy to enhance therapeutic efficacy and overcome resistance, often through synergistic mechanisms that increase tumor immunogenicity or susceptibility to immune-mediated attack [71]. Collectively, these findings support the relevance of specific PBMCs phenotypes as indicators of therapeutic response.

Beyond their role in predicting therapeutic efficacy, PBMC immunophenotypes may also provide valuable insights into the development of immune-related adverse events (irAEs) associated with immunotherapy [38]. Emerging evidence suggests that heightened systemic immune activation, expansion of specific T cell subsets, and dysregulated checkpoint expression may predispose patients to autoimmune toxicity. Therefore, longitudinal monitoring of PBMC profiles could help identify patients at risk of irAEs and guide treatment adjustment.

In the context of ICI therapy, resistance has been strongly associated with systemic immune dysfunction reflected in PBMC phenotypes. Expanded regulatory T cell populations, accumulation of immunosuppressive myeloid cells such as MDSCs, and persistent expression of inhibitory receptors including PD-1, TIGIT, and TIM-3 on circulating T cells contribute to impaired antitumor immunity and reduced responsiveness to ICIs. In addition, chronic antigen stimulation may promote progressive T cell exhaustion, characterized by diminished cytotoxic activity and defective cytokine production [3,72,73]. These mechanisms collectively contribute to the establishment of an immunosuppressive systemic environment that limits effective immune reactivation during checkpoint blockade therapy.

Distinct PBMC phenotypic alterations have also been associated with resistance to chemotherapy and other conventional anticancer treatments. In particular, polarization of circulating monocytes toward anti-inflammatory or tumor-supportive phenotypes, together with increased production of suppressive cytokines such as IL-10 and TGF-β, may contribute to reduced treatment efficacy by promoting immune tolerance and tissue remodeling [10,74,75]. Chemotherapy-induced immune reprogramming can additionally alter the balance between effector and suppressive immune cell subsets, thereby facilitating tumor persistence and disease progression. These observations suggest that PBMC immunophenotypes may serve not only as biomarkers of treatment response but also as indicators of therapy-specific resistance mechanisms.

Taken together, these observations highlight both the potential and the current limitations of PBMC-based biomarkers. From a translational perspective, PBMC immunophenotyping represents a minimally invasive and dynamic tool with significant clinical potential. Its application could support patient stratification, real-time monitoring of treatment response, and early identification of resistance mechanisms. However, its clinical implementation requires overcoming current limitations, including variability in analytical platforms, lack of standardized protocols, and limited validation in large, multicenter cohorts. Future research should prioritize the integration of PBMC immunophenotyping with high-dimensional multi-omics approaches and advanced computational methods. In particular, the combined application of single-cell RNA sequencing and flow cytometry-based immunophenotyping may enable simultaneous characterization of immune cell transcriptomic profiles and surface protein expression, thereby improving the resolution and functional interpretation of PBMC subsets. In parallel, machine learning algorithms may facilitate the integration of multidimensional immune datasets and support the development of predictive models for treatment response, resistance, and immune-related adverse events. Artificial intelligence, particularly machine learning and deep learning techniques, offers promising solutions by enabling the analysis of complex and multidimensional data to predict treatment outcomes with greater accuracy [76,77]. Furthermore, emerging spatial transcriptomics technologies could help validate the migration and localization of circulating immune cell subsets within the tumor microenvironment, providing additional insight into PBMC–tumor interactions and systemic immune dynamics [78,79].

As highlighted in this review, distinct immune modulations are linked to treatment response or tumor progression and have been associated with the PBMC immunophenotype, conferring prognostic and predictive relevance [9]. Despite growing interest in PBMC immunophenotyping, its clinical translation remains limited by substantial heterogeneity across studies. This includes technical variability (e.g., flow cytometry panel design, sample processing protocols, and timing of blood collection), clinical differences (e.g., treatment line, prior therapies, tumor burden, tumor stage, and comorbidities), and inconsistencies in data analysis, all of which hinder reproducibility and cross-study comparability. Addressing these challenges will require the implementation of standardized immunophenotyping protocols in clinical trials and the validation of candidate biomarkers in large, multi-center cohorts. Furthermore, future research should explore biomarker-driven patient stratification strategies and combinatorial therapeutic approaches targeting specific immune cell subsets to overcome treatment resistance. Future studies integrating high-dimensional approaches such as single-cell RNA sequencing, cytokine profiling, and functional assays will be essential to refine PBMC-based biomarkers and uncover underlying mechanisms. Importantly, the relationship between PBMC immune phenotypes and irAEs remains insufficiently characterized and represents a critical area for investigation, given its potential to inform both treatment efficacy and toxicity.

The findings reported here suggest that characterization of circulating immune cell subsets in cancer is relevant, as systemic immune dysfunction can influence antitumor immunity, treatment efficacy, and the development of autoimmunity. Furthermore, the development of prognostic and predictive therapy-related biomarkers is more efficient when candidates are identified from peripheral blood, as this type of sample allows for routine monitoring. Comprehensive characterization of the systemic immune landscape in cancer thus holds significant promise for advancing the identification of clinically relevant immunological biomarkers and improving personalized cancer therapy [29].

Importantly, most currently available studies are observational and primarily demonstrate correlations between PBMC phenotypes and treatment response or disease progression, whereas robust causal evidence from interventional studies or Mendelian randomization approaches remains largely lacking. Establishing causal relationships between systemic immune phenotypes and therapeutic resistance therefore remains a major unresolved challenge and an important priority for future research.

## 7 Conclusions

PBMC immunophenotyping represents a compelling approach for capturing systemic immune dynamics in cancer through minimally invasive sampling. Across tumor types, consistent evidence supports its association with clinically relevant processes, including response to immunotherapy, disease progression, and immune-related toxicity. However, the field has not yet reached the level of robustness required for routine clinical implementation.

The main barrier is not the lack of promising signals, but the absence of standardization and validation. Current studies remain limited by heterogeneous methodologies, small and non-comparable cohorts, and insufficient longitudinal designs. As a result, many proposed immune signatures, such as exhausted CD8^+^ T cell subsets, regulatory T cell expansion, or myeloid-derived suppressor cell accumulation, lack reproducibility across settings and cannot yet be reliably translated into clinical decision-making tools.

A key unmet need is the establishment of harmonized frameworks that enable cross-study comparability and distinguish universal immune patterns from tumor-specific signatures. Addressing this will be essential to move from descriptive associations to actionable biomarkers.

From a translational perspective, the integration of PBMC immunophenotyping with multi-omics approaches and advanced computational models offers a realistic path forward. These strategies may enhance predictive accuracy, support patient stratification, and enable dynamic monitoring of treatment response.

Ultimately, the clinical value of PBMC-based biomarkers will depend on rigorous validation in well-designed prospective studies. Bridging this gap will determine whether PBMC immunophenotyping can evolve from a promising research tool into a reliable component of precision oncology.

## Data Availability

Not applicable.
